# An Inverse Problem Solution Scheme for Solving the Optimization Problem of Drug-Controlled Release from Multilaminated Devices

**DOI:** 10.1155/2020/8380691

**Published:** 2020-08-01

**Authors:** Xinming Zhang

**Affiliations:** School of Science, Harbin Institute of Technology (Shenzhen), Shenzhen 518055, China

## Abstract

The optimization problem of drug release based on the multilaminated drug-controlled release devices has been solved in this paper under the inverse problem solution scheme. From the viewpoint of inverse problem, the solution of optimization problem can be regarded as the solution problem of a Fredholm integral equation of first kind. The solution of the Fredholm integral equation of first kind is a well-known ill-posed problem. In order to solve the severe ill-posedness, a modified regularization method is presented based on the Tikhonov regularization method and the truncated singular value decomposition method. The convergence analysis of the modified regularization method is also given. The optimization results of the initial drug concentration distribution obtained by the modified regularization method demonstrate that the inverse problem solution scheme proposed in this paper has the advantages of the numerical accuracy and antinoise property.

## 1. Introduction

It is well known that the controlled release device is usually used to regulate the release of active material for maintaining a preset concentration of the active material for a specified period of time. In recent years, the technique has been widely used in many fields, including drug, food, and cosmetics [1, 2], due to its safety and effectiveness. Especially, in the drug research field, the controlled release device has been of considerable interest to many scholars. Generally, the burst effect is not a desired delivery profile for drug release behavior, which will cause overdose of drug and systemic toxicity [3]. During the last three decades, several matrix geometries have been used to avoid this undesired effect, in which, the multilayer matrix device is an effective and simple device geometry[4–6]. For the multilayer matrix devices, the matrix core, containing the drug, is covered by one or more modulating layers that act as rate-controlling barriers. Researches have revealed that for the multilayer matrix devices, the reasonable initial drug loading can efficiently control the burst effect [7].

For understanding and simulating the real release mechanisms from these complicated multilaminated matrix devices better, application of mathematical models is a good choice [8–10]. In 1961, Higuchi [11], who is regarded as the “father of mathematical modeling of drug release”, proposed a simple but surprising equation to quantify the drug release from monolithic dispersions with slab geometry: the famous Higuchi equation. After that, more and more scientists paid their attentions to the mathematical modeling of drug release, which can significantly contribute to the product development and mechanism understanding. Especially, over the last thirty years, there are many new progresses for different controlled release devices. Helbling et al. [12] used the refined integral method to study the controlled dispersed drug release from planar nonerodible polymeric matrices and derived an analytical solution in 2010 and then presented a novel mathematical model for drug-controlled release based on the torus-shaped single-layer devices in 2011. Streubel et al. [13] developed a new multilayer matrix tablets to obtain the bimodal drug release profiles and used theophylline and acetaminophen as the model drugs to investigate the effects of some parameters on the resulting release rates. Yin and Li [14] introduced the fractional calculus to model the anomalous diffusions and presented some new mathematical models to describe the drug release based on degradable and nondegradable slab matrices. Wu and Zhou [15] studied several factors affecting the kinetics of diffusional drug release based on the finite element method. Nevertheless, most of the previous methods focus on how to forecast the drug release profiles based on the given parameters. There have been few studies to investigate how to choose the suitable control parameters, in multilayer devices, to make the drug release profile as close to the desired release profile as possible. As far as we known, in 1998 and 1999, articles [5, 16] made relatively big contribution to this area firstly. In these two manuscripts, Lu and his coworkers employed a formal optimization approach to correctly determine the initial drug concentration in the layer so as to coincide with the required release profile as much as possible. To obtain the desired release rates in multilaminated drug delivery devices, Georgiadis and Kostoglou [7] presented a systematic optimization framework based on a simple mathematical model. Nauman et al. [17] designed a multilaminated drug delivery device, which has two or three layers with different initial drug concentration distributions. By adjusting the parameters in this device, all kinds of drug release profiles can be obtained. As shown in previous articles, the optimizations of the available control parameters have been performed in the frame of optimization. However, we can also treat this problem from another viewpoint, that is, the viewpoint of inverse problem. In fact, many practical problems in different fields can be reduced into the solution of inversion problems, such as geophysical exploration and medical imaging. In this article, from the viewpoint of the inverse problem, we adopt a solution scheme of inverse problem to solve the optimization problem of multilaminated drug-controlled release, which has been transformed into a diffusion equation initial value inverse problem. A classical regularization method, that is Tikhonov regularization method [18], and its variant have been attempted to solve the inverse problem. In 1963, Tikhonov proposed the Tikhonov regularization method firstly. Since then, many scholars turned their attentions to this rather effective method, developed many different variants in different space settings, and applied these regularization methods to solve all kinds of problems in science and engineering fields [19–22]. Fu et al. [23] applied the Fourier method to solve some ill-posed problems and systematically considered a posteriori choice of the regularization parameter; Zheng and Wei [24] used a spectral regularization method to solve the Cauchy problem of TFADE based on the solution given by the Fourier method; Zhao [25] introduced a mollification method to solve the ill-posed problem by using the expanded Hermit functions; Cheng et al. [26] presented an optimal filtering method to approximate a Cauchy problem for the Helmholtz equation in a rectangle and showed the Hölder-type error estimate; Bonesky et al. [27] applied an adaptive wavelet algorithms to solve an inverse parabolic problem describing the industrial process of melting iron ore in a steel furnace; Zhang and Li [28] established a new regularization method to solve the ill-posed problem based on the singular system theory of compact operator.

In this article, we propose a modified regularization method based on a new regularizing filter function obtained by combining the method presented in the paper [28] with the truncated singular value decomposition method. We also show the convergence analysis of the proposed method. The regularization parameters are determined with the L-curve method suggested by Hansen in [29]. Then, we apply the modified regularization method to the optimization problem of the initial drug concentration distribution based on the multilaminated drug-controlled release devices and obtain some better results.

In the reminder of the paper, it is organized as follows. We describe the mathematical model for the multilaminated drug-controlled release devices and the corresponding inverse problem solution scheme for the optimization of drug-controlled release in Section 2. In Section 3, a modified regularization method is described in detail. This is followed by numerical simulation in Section 4 and Section 5, and lastly, the conclusion is indicated in Section 6.

## 2. Mathematical Model and Inverse Problem Solution Scheme

### 2.1. Mathematical Model

**Figure 1:**
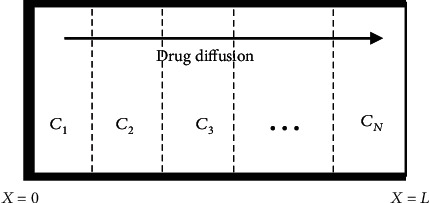
Drug release from a multilaminated drug-controlled release device.

Figure 1 shows a multilaminated drug-controlled release device, which has *N* layers. The device has a thickness *L* and initial drug concentration *V*(*X*). It is sealed at the leftmost side by an impermeable layer and contacts with outside through the rightmost side. It is assumed that the device is not significantly swelling and eroding during drug release. Here, C_*i*_(*i* = 1, 2, ⋯, *N*) are the drug concentrations of each layer, respectively. The present analysis focuses only on the case of low drug concentration.

Mathematically, this problem can be modeled as one-dimensional partial differential equation based on Fick's second law of diffusion:

(1) $$\begin{array}{c} \frac{\partial C}{\partial \tau }=\frac{\partial }{\partial X}\left(D\frac{\partial C}{\partial X}\right), \end{array}$$

where *C* is the drug concentration, *D* is the diffusivity, and *X* and *τ* are the position and release time for one-dimensional diffusional processes, respectively.

Under the assumption that zero flux and zero concentration are prescribed at the interface with impermeable layer and the environment, respectively, thus, the boundary conditions are as follows:

(2) $$\begin{array}{c} {\left.\frac{\partial C}{\partial X}\right|}_{X=0}=0,\;\tau >0, \end{array}$$

(3) $$\begin{array}{c} C\left(\tau ,L\right)=0,\;\tau >0. \end{array}$$

The initial conditions are imposed as follows:

(4) $$\begin{array}{c} C\left(0,X\right)=V\left(X\right),\tau =0,\;0<X<L. \end{array}$$

The flux of drug is also defined as follows:

(5) $$\begin{array}{c} J\left(\tau ,L\right)=-D_{\left(X=L\right)}{\left.\frac{\partial C}{\partial X}\right|}_{X=L},\;\tau >0, \end{array}$$

where *V*(*X*) denotes the initial drug concentration.

### 2.2. The Inverse Problem Solution Scheme

For the mathematical model (Equations (1)–(5)), if the initial conditions and the boundary conditions are known, the process to compute the concentration distribute functions *C*(*τ*, *X*) is a forward problem, which is a well-posed problem. Conversely, how to identify the initial Condition (4) is a classical inverse problem, if we know the boundary Conditions (2) and (3) and additional Condition (5). Generally speaking, the inverse problem is ill-posed and always needs to be solved with some special algorithms, e.g., the Tikhonov regularization method.

Assume that the diffusivity is constant, we use the following dimensionless processing to simplify the computation process: *c* = *C*/*C*_0_, *v* = *V*(*X*)/*C*_0_, *x* = *X*/*L*, *t* = *D*_0_*τ*/*L*^2^, *j* = *JL*/*D*_0_*C*_0_, *d* = *D*/*D*_0_ = 1, where *C*_0_ is a reference concentration and *D*_0_ is a reference diffusivity.

Thus, the previous mathematical model (Equations (1)–(5)) can be rewritten as follows:

(6) $$\begin{array}{c} \frac{\partial c}{\partial t}=\frac{\partial^{2}c}{\partial x^{2}}. \end{array}$$

Boundary conditions:

(7) $$\begin{array}{c} {\left.\frac{\partial c}{\partial x}\right|}_{x=0}=0,t>0,\\ c\left(t,1\right)=0,t>0. \end{array}$$

Initial conditions:

(8) $$\begin{array}{c} c\left(0,x\right)=v\left(x\right),t=0,0<x<1, \end{array}$$

Additional conditions:

(9) $$\begin{array}{c} j\left(t,1\right)={\left.-\frac{\partial c}{\partial x}\right|}_{x=1}=j\left(t\right),t>0. \end{array}$$

In fact, from the viewpoint of inverse problem, the problem to determine the initial conditions *v*(*t*) based on the above mathematical model (Equations (6)–(9)) is a diffusion equation inverse problem and can further come down to a solution problem of Fredholm integral equation of first kind.

The first step is to solve Equations (6)–(8), which is a diffusion equation initial boundary value problem. The method of separating variables leads to the analytical solution:

(10) $$\begin{array}{c} c\left(t,x\right)=\overset{\infty }{\underset{k=0}{\sum }}2e^{-{\left[\left(k+1/2\right)\pi \right]}^{2}t}\cos\left(k+\frac{1}{2}\right)\pi x\int_{0}^{1}v\left(x\right)\cos\left(k+\frac{1}{2}\right)\pi xdx. \end{array}$$

From Equation (10), the flux *j*(*t*), namely, the additional conditions, in Equation (9), can be determined by differentiation with respect to *x* as follows:

(11) $$\begin{array}{c} j\left(t\right)=-{\left.\frac{\partial c}{\partial x}\right|}_{x=1}=\overset{\infty }{\underset{k=0}{\sum }}2e^{-{\left[\left(k+1/2\right)\pi \right]}^{2}t}\left(k+\frac{1}{2}\right)\pi \sin\left(k+\frac{1}{2}\right)\pi x\int_{0}^{1}v\left(x\right)\cos\left(k+\frac{1}{2}\right)\pi xdx=\overset{\infty }{\underset{k=0}{\sum }}2{\left(-1\right)}^{k}\left(k+\frac{1}{2}\right)\pi e^{-{\left[\left(k+1/2\right)\pi \right]}^{2}t}\int_{0}^{1}v\left(x\right)\cos\left(k+\frac{1}{2}\right)\pi xdx=2\int_{0}^{1}\left[\overset{\infty }{\underset{k=0}{\sum }}{\left(-1\right)}^{k}\left(k+\frac{1}{2}\right)\pi e^{-{\left[\left(k+1/2\right)\pi \right]}^{2}t}\cos\left(k+\frac{1}{2}\right)\pi x\right]v\left(x\right)dx. \end{array}$$

Then, using Equation (11), the previous inverse problem (Equations (6)–(9)) for determining initial conditions can further be transformed into the following Fredholm integral equation of first kind:

(12) $$\begin{array}{c} 2\int_{0}^{1}\left[\overset{\infty }{\underset{k=0}{\sum }}{\left(-1\right)}^{k}\left(k+\frac{1}{2}\right)\pi e^{-{\left[\left(k+1/2\right)\pi \right]}^{2}t}\cos\left(k+\frac{1}{2}\right)\pi x\right]v\left(x\right)dx=j\left(t\right), \end{array}$$

where *v*(*x*) is the unknown function and the kernel function is

(13) $$\begin{array}{c} k\left(x,t\right)=\overset{\infty }{\underset{k=0}{\sum }}{\left(-1\right)}^{k}\left(k+\frac{1}{2}\right)\pi e^{-{\left[\left(k+1/2\right)\pi \right]}^{2}t}\cos\left(k+\frac{1}{2}\right)\pi x. \end{array}$$

Thus, successful solution of the Fredholm integral equation of first kind will lead to the effective identification of the initial conditions in the mathematical model (Equations (6)–(9)), that is, the optimization of the initial drug concentration in the optimization problem of drug release based on the multilaminated drug-controlled release devices. However, the solution of the Fredholm integral equation of first kind is a well-known ill-posed problem, especially, for the case of input data with noise. We usually need a suitable and specific method to solve it. In the next section, we propose a new regularization method to solve Equation (12).

## 3. The Modified Regularization Method

Kirsch [30] proposed the concept of regularizing filter function to investigate the ill-posed problem. As shown in [30], for the regularizing filter function, we have the following Lemma:

**Lemma 1 .**

Let *X* and *Y* be both Hilbert spaces, *K* : *X* → *Y* be compact with singular system (*μ*_*i*_, *x*_*i*_, *y*_*i*_), and *q* : (0, +∞) × (0, ‖*K*‖] → *R* be a function with the following properties:

1. ∣*q*(*α*, *μ*) | ≤1, ∀*α* ∈ (0, +∞), ∀*μ* ∈ (0, ‖*K*‖]
2. For ∀*α* ∈ (0, +∞), there exists *c*(*α*) > 0, such that ∣*q*(*α*, *μ*) | ≤*c*(*α*)*μ*,∀*μ* ∈ (0, ‖*K*‖]
3. $\underset{\alpha \to 0}{\lim}q\left(\alpha ,\mu \right)=1$, ∀*μ* ∈ (0, ‖*K*‖]

Then, the operator *R*_*α*_ : *Y* → *X*is defined by

(14) $$\begin{array}{c} R_{\alpha }y=\overset{\infty }{\underset{i=1}{\sum }}\frac{q\left(\alpha ,\mu_{i}\right)}{\mu_{i}}\left(y,y_{i}\right)x_{i}, \end{array}$$

which is a regularization strategy, where ‖*R*_*α*_‖ ≤ *c*(*α*). We call the function *q*(*α*, *μ*) satisfying the previous three properties regularizing filter function for *K*.

Based on the singular system theory of the compact operator, the regularized solution of the famous Tikhonov regularization method can be expressed in the following formula:

(15) $$\begin{array}{c} x_{\alpha }^{\delta }=R_{\alpha }y_{\delta }=\overset{\infty }{\underset{i=1}{\sum }}\frac{q\left(\alpha ,\mu_{i}\right)}{\mu_{i}}\left(y_{\delta },y_{i}\right)x_{i}, \end{array}$$

where *q*(*α*, *μ*) = *μ*^2^/(*α* + *μ*^2^), (*α* > 0, 0 < *μ* ≤ ‖*K*‖) is the Tikhonov regularizing filter function, and *y*_*δ*_ is the disturbed data.

In the paper [28], Zhang and Li presented an improved regularization method based on a new regularizing filter function *q*(*α*, *μ*) = *μ*^*σ*^/(*α* + *μ*^*σr*^)^1/*r*^,  *r* > 0,   ∈ *σ* ≥ 1 and proved that the regularization solution can achieve the optimal asymptotic convergence rate by selecting reasonable regularization parameters.

Similarly, in this paper, a new regularization method is introduced based on the previous improved regularization method and the truncated singular value decomposition (TSVD) regularization method. The convergence and the optimal asymptotic order of the new regularized solution are also obtained.

### 3.1. A New Regularizing Filter Function

The regularizing filter function corresponding to the improved regularization method proposed in literature [28] is as follows:

(16) $$\begin{array}{c} q\left(\alpha ,\mu \right)=\frac{\mu^{\sigma }}{{\left(\alpha +\mu^{\sigma r}\right)}^{1/r}},\;r>0,\;\sigma \geq 1. \end{array}$$

The filter function of the truncated singular value decomposition regularization method is as follows:

(17) $$\begin{array}{c} q\left(\alpha ,\mu \right)=\left\{\begin{array}{cc} 1 & \mu \geq \alpha \\ 0 & \mu <\alpha \end{array}.\right. \end{array}$$

Based on the previous two methods, we introduce a new regularizing filter function, which is defined as follows:

(18) $$\begin{array}{c} q\left(\alpha ,\mu \right)=\left\{\begin{array}{cc} 1 & \mu^{\sigma r}\geq \alpha \\ \frac{\mu^{\sigma }}{{\left(\alpha +\mu^{\sigma r}\right)}^{1/r}} & \mu^{\sigma r}<\alpha \end{array},\right. \end{array}$$

where *α* > 0,  0 < *μ* ≤ ‖*K*‖,  *r* > 0,  *σ* ≥ 1.

Using Equation (18), we can make the large singular value not be modified and the small singular value be filtered. Thus, the modified regularization method adopting the new regularizing filter function will lead to a more accurate regularized solution.

For the new regularizing filter function, we prove the following theorem.

**Theorem 1 .**

The function **q**(*α*, *μ*) defined by Equation (18) satisfies the assumptions (1), (2), and (3) of **Lemma**1, namely, the function **q**(*α*, *μ*) defined by Equation (18) is a regularizing filter function.

**Proof**

1. It is sufficient to consider the case *μ*^*σr*^ < *α*. In this case, *q*(*α*, *μ*) = *μ*^*σ*^/(*α* + *μ*^*σr*^)^1/*r*^ < 1 because *μ*^*σ*^ = (*μ*^*σr*^)^1/*r*^ < (*α* + *μ*^*σr*^)^1/*r*^
2. For the case of *μ*^*σr*^ ≥ *α*, we conclude that *μ*^*σr*^/*α* ≥ 1, that is, *μ*/*α*^1/*σr*^ ≥ 1; this proves that |*q*(*α*, *μ*)| = 1 ≤ 1/(*α*)^1/*σr*^*μ*

For the case of *μ*^*σr*^ < *α* , we have *q*(*α*, *μ*) = *μ*^*σ*^/(*α* + *μ*^*σr*^)^1/*r*^.

The choice *q* = *σ*/*σ* − 1 leads to

(19) $$\begin{array}{c} q\left(\alpha ,\mu \right)=\frac{\mu }{{\left(\alpha +\mu^{r}\right)}^{1/r}}\leq \frac{\mu }{\alpha^{1/r}}. \end{array}$$

If *σ* > 1, let *p* = *σ* and *q* = *σ*/*σ* − 1. Thanks to the Yong inequality *α* + *μ*^*σr*^ ≥ *α*^1/*p*^ · *μ*^*σr*/*q*^, we have *α* + *μ*^*σr*^ ≥ *α*^1/*σ*^ · *μ*^*r*(*σ* − 1)^, which yields

(20) $$\begin{array}{c} \frac{\mu^{\sigma }}{{\left(\alpha +\mu^{\sigma r}\right)}^{1/r}}\leq \frac{\mu^{\sigma }}{\alpha^{1/\sigma r}\cdot \mu^{\sigma -1}}=\frac{\mu }{\alpha^{1/\sigma r}}. \end{array}$$

Thus, the inequality *q*(*α*, *μ*) ≤ *μ*/*α*^1/*σr*^ holds for ∀*σ* ≥ 1. That is the property (2) in Lemma 1 holds with*c*(*α*) = 1/*α*^1/*σr*^, for∀*α* > 0.

(3) It is obvious that *q*(*α*, *μ*) = 1, as *α* → 0

So, from **Lemma**1, we can know that the function **q**(*α*, *μ*) defined by Equation (18) is a regularizing filter function and the corresponding regularization operator *R*_*α*_ : *Y* → *X* is as follows:

(21) $$\begin{array}{c} R_{\alpha }y=\overset{\infty }{\underset{i=1}{\sum }}\frac{q\left(\alpha ,\mu_{i}\right)}{\mu_{i}}\left(y,y_{i}\right)x_{i}. \end{array}$$

Then, a modified regularization method can be constructed based on the regularization operator *R*_*α*_ from Equation (21), which can also be used to solve the ill-posed problem for the case of input data with noise. The regularized solution *x*_*α*_^*δ*^ is therefore defined by *x*_*α*_^*δ*^ = *R*_*α*_*y*_*δ*_ = ∑_*i*=1_^∞^(*q*(*α*, *μ*_*i*_)/*μ*_*i*_)(*y*_*δ*_, *y*_*i*_)*x*_*i*_.

We combine the singular system theory of the compact operator with the previous theorem and show the following result for the regularized solution.

### 3.2. Error Analysis of the Regularized Solution

**Theorem 2 .**

Let *x*^+^ = (*K*^∗^*K*)^*v*^*z* ∈ *R*(*K*^∗^*K*)^*v*^, *z* ∈ *X*, with ‖*z*‖ ≤ *E*; for the regularization operators *R*_*α*_ : *Y* → *X* from Equation (21), we choose the regularized parameters *α*(*δ*) = *c*(*δ*/*E*)^*σr*/(2*v* + 1)^ for some *c* > 0; then, the following estimate holds:

(22) $$\begin{array}{c} \left\|x_{\alpha \left(\delta \right)}^{\delta }-x^{+}\right\|=O\left(\delta^{2v/\left(2v+1\right)}\right), \end{array}$$

where *x*^+^ is the solution of *Kx* = *y*, *x*_*α*(*δ*)_^*δ*^≔*R*_*α*_*y*^*δ*^ is the approximation of *x*, and *K*^∗^ denotes the adjoint operator of *K*.

**Proof**

The error between *x* and *x*_*α*(*δ*)_^*δ*^ is

(23) $$\begin{array}{c} \left\|x_{\alpha }^{\delta }-x^{+}\right\|\;\leq \left\|R_{\alpha }\right\|\cdot \delta +\left\|R_{\alpha }y-x^{+}\right\|, \end{array}$$

**Theorem** 1 yields ‖*R*_*α*_‖ ≤ *c*(*α*) = 1/*α*^1/*σr*^. From *R*_*α*_*Kx* = ∑_*i*=1_^∞^(*q*(*α*, *μ*_*i*_)/*μ*_*i*_)(*Kx*, *y*_*i*_)*x*_*i*_, *x* = ∑_*i*=1_^∞^(*x*, *x*_*i*_)*x*_*i*_, and (*Kx*, *y*_*i*_) = (*x*, *K*^∗^*y*_*i*_) = *μ*_*i*_(*x*, *x*_*i*_), we conclude that

(24) $$\begin{array}{c} {\left\|R_{\alpha }y-x^{+}\right\|}^{2}=\overset{\infty }{\underset{i=1}{\sum }}{\left|q\left(\alpha ,\mu_{i}\right)-1\right|}^{2}\cdot {\left|\left(x^{+},x_{i}\right)\right|}^{2}=\overset{\infty }{\underset{i=1}{\sum }}{\left|q\left(\alpha ,\mu_{i}\right)-1\right|}^{2}\cdot {\left|\left({\left(K^{*}K\right)}^{v}z,x_{i}\right)\right|}^{2}=\overset{\infty }{\underset{i=1}{\sum }}{\left|q\left(\alpha ,\mu_{i}\right)-1\right|}^{2}\cdot {\left|\left(\overset{\infty }{\underset{j=1}{\sum }}\mu_{j}^{2v}\left(z,x_{j}\right)x_{j},x_{i}\right)\right|}^{2}=\overset{\infty }{\underset{i=1}{\sum }}{\left|q\left(\alpha ,\mu_{i}\right)-1\right|}^{2}\cdot {\left|\mu_{i}^{2v}\left(z,x_{i}\right)\right|}^{2}=\overset{\infty }{\underset{i=1}{\sum }}{\left|q\left(\alpha ,\mu_{i}\right)-1\right|}^{2}\cdot \mu_{i}^{4v}\cdot {\left|\left(z,x_{i}\right)\right|}^{2}. \end{array}$$

If *μ*^*σr*^ < *α*, |*q*(*α*, *μ*_*i*_) − 1| < 1 because 0 < *q*(*α*, *μ*) < 1; thus,

(25) $$\begin{array}{c} \left|q\left(\alpha ,\mu_{i}\right)-1\right|\cdot \mu_{i}^{2v}<\mu_{i}^{2v}={\left(\mu_{i}^{\sigma r}\right)}^{2v/\sigma r}<\alpha^{2v/\sigma r}, \end{array}$$

If *μ*^*σr*^ ≥ *α*, *q*(*α*, *μ*) = 1, so

(26) $$\begin{array}{c} \left|q\left(\alpha ,\mu_{i}\right)-1\right|\cdot \mu_{i}^{2v}=0\cdot \mu_{i}^{2v}=0<\alpha^{2v/\sigma r}. \end{array}$$

Then, |*q*(*α*, *μ*_*i*_) − 1| · *μ*_*i*_^2*v*^ < *α*^2*v*/*σr*^ holds for all two cases.

Therefore,

(27) $$\begin{array}{c} {\left\|R_{\alpha }y-x^{+}\right\|}^{2}<\alpha^{4v/\sigma r}\overset{\infty }{\underset{i=1}{\sum }}{\left|\left(z,x_{i}\right)\right|}^{2}=\alpha^{4v/\sigma r}\cdot {\left\|z\right\|}^{2}\leq \alpha^{4v/\sigma r}\cdot E^{2}, \end{array}$$

that is ‖*R*_*α*_*y* − *x*^+^‖ < *α*^2*v*/*σr*^ · *E*. Thus, we have shown that

(28) $$\begin{array}{c} \left\|x_{\alpha }^{\delta }-x^{+}\right\|\leq \frac{1}{\alpha^{1/\sigma r}}\cdot \delta +\alpha^{2v/\sigma r}\cdot E. \end{array}$$

The choice of *α*(*δ*) = *c*(*δ*/*E*)^*σr*/(2*v* + 1)^ leads to the corresponding estimate

(29) $$\begin{array}{c} \left\|x_{\alpha \left(\delta \right)}^{\delta }-x^{+}\right\|\leq \delta \cdot {\left[c{\left(\frac{\delta }{E}\right)}^{\sigma r/\left(2v+1\right)}\right]}^{-1/\sigma r}+{\left[c{\left(\frac{\delta }{E}\right)}^{\sigma r/\left(2v+1\right)}\right]}^{2v/\sigma r}\cdot E=\delta \cdot \left[c^{-1/\sigma r}\cdot {\left(\frac{\delta }{E}\right)}^{-1/\left(2v+1\right)}\right]+c^{2v/\sigma r}\cdot {\left(\frac{\delta }{E}\right)}^{2v/\left(2v+1\right)}\cdot E=c^{-1/\sigma r}\cdot E^{1/\left(2v+1\right)}\cdot \delta^{1-\left(1/\left(2v+1\right)\right)}+c^{2v/\sigma r}\cdot \delta^{2v/\left(2v+1\right)}\cdot E^{1-\left(2v/\left(2v+1\right)\right)}=\left(c^{-1/\sigma r}+c^{2v/\sigma r}\right)\cdot E^{1/\left(2v+1\right)}\cdot \delta^{2v/\left(2v+1\right)}. \end{array}$$

This yields

(30) $$\begin{array}{c} \left\|x_{\alpha \left(\delta \right)}^{\delta }-x^{+}\right\|=O\left(\delta^{2v/\left(2v+1\right)}\right). \end{array}$$

## 4. Solution of the Fredholm Integral Equation of First Kind

For validation purpose, in this section, the modified regularization method proposed in this paper is applied to solve the Fredholm integral equation of first kind.

**Example 1 .**

We first consider the following integral equation of first kind.

(31) $$\begin{array}{c} \int_{0}^{1}\left(1+ts\right)e^{ts}x\left(s\right)ds=y\left(t\right),\;0\leq t\leq 1, \end{array}$$

with the right-hand side and the analysis solution given by *y*(*t*) = *e*^*t*^ and *x*(*t*) = 1.

We discretize the integral equation by the compound trapezoidal formula and obtain the linear system.

(32) $$\begin{array}{c} AX=b, \end{array}$$

where **A** ∈ *R*^100×100^ and the error-free right-hand side **b** = [*y*(*t*_0_),⋯,*y*(*t*_*m*_)]^T^ ∈ *R*^100^. The associated contaminated vector $\tilde{b}$ is given by the following formula.

(33) $$\begin{array}{c} \tilde{b}=b\left(1+\delta \times \mathrm{rand}\right), \end{array}$$

where *δ* denotes the noise level and rand is a number randomly generated within the interval [0, 1]. In this example, the noise level is assumed to be 0.001.

**Figure 2:**
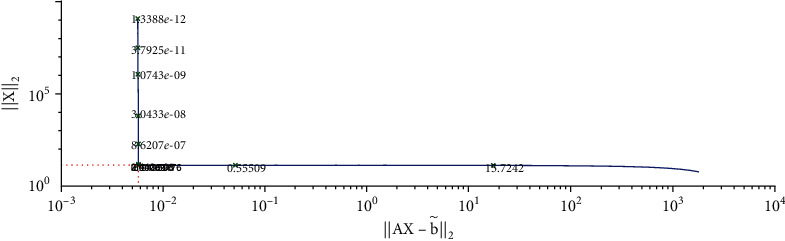
Regularization parameter choice (L-curve).

**Figure 3:**
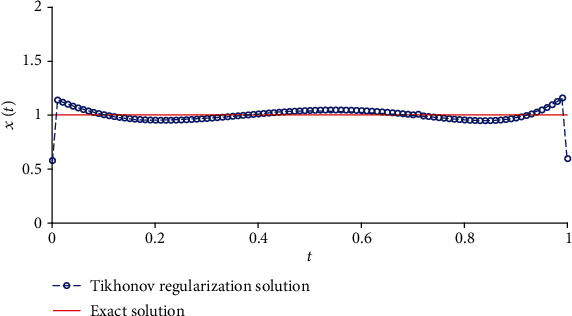
The results of Tikhonov regularization method.

**Figure 4:**
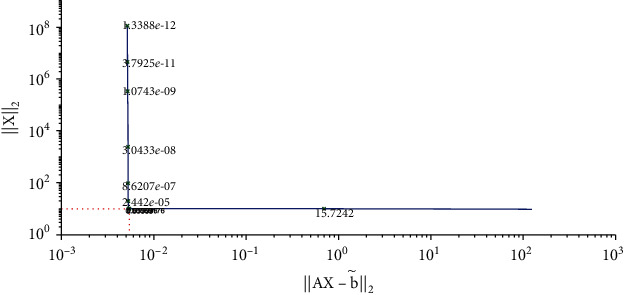
Regularization parameter choice (L-curve).

**Figure 5:**
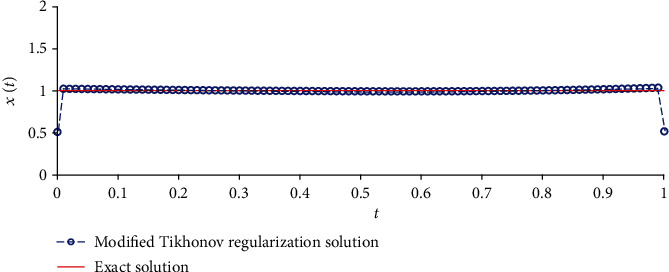
The results of modified regularization method.

We solve equation $AX=\tilde{b}$ with the Tikhonov regularization method and our method, respectively. The regularization parameters are chosen by using the L-curve method. The corresponding L-curve for the Tikhonov regularization method is shown in Figure 2. The corner of L-curve is located at the points (${\left\|AX-\tilde{b}\right\|}_{2}$, ‖**X**‖_2_) with the regularization parameter *α* = 3.7866 × 10^−3^. We set the parameters of the modified regularization method *σ* = 4 and *r* = 1.5 as suggested in the paper [28].  Figure 3 depicts the comparison between the results obtained by the classical Tikhonov regularization method and the exact solution.  Figure 4 shows the corresponding L-curve for the modified regularization method, and the regularization parameter is 2.1147^∗^*e* − 3. And we compare the results obtained by the modified regularization method and the exact solution in Figure 5. As we can see that, from these two results, the modified regularization method is more effective than the classical Tikhonov regularization method.

**Example 2 .**

Consider the following Fredholm integral equation of first kind.

(34) $$\begin{array}{c} \int_{0}^{1}e^{ts}x\left(s\right)ds=y\left(t\right),\;0\leq t\leq 1, \end{array}$$

which has a unique exact solution *x*(*t*) = *e*^2*t*^, and the right-hand side is given by *y*(*t*) = (*e*^*t*+2^ − 1)/(*t* + 2).

**Figure 6:**
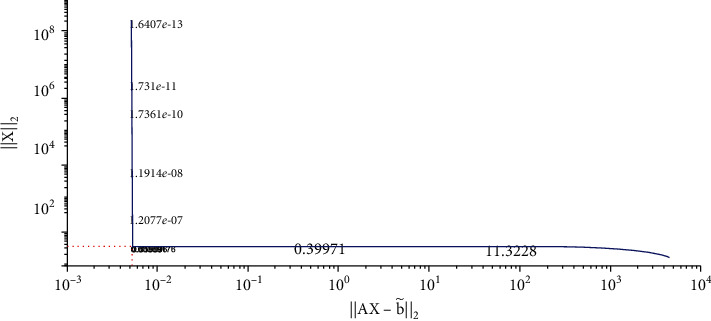
Regularization parameter choice (L-curve).

**Figure 7:**
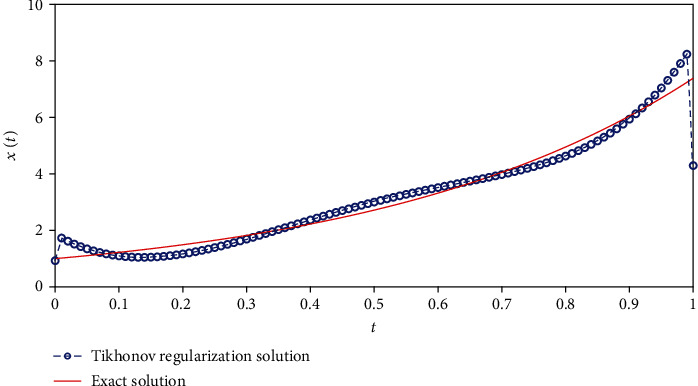
The results of Tikhonov regularization method.

**Figure 8:**
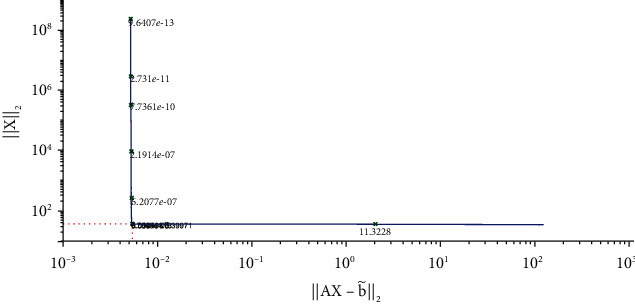
Regularization parameter choice (L-curve).

**Figure 9:**
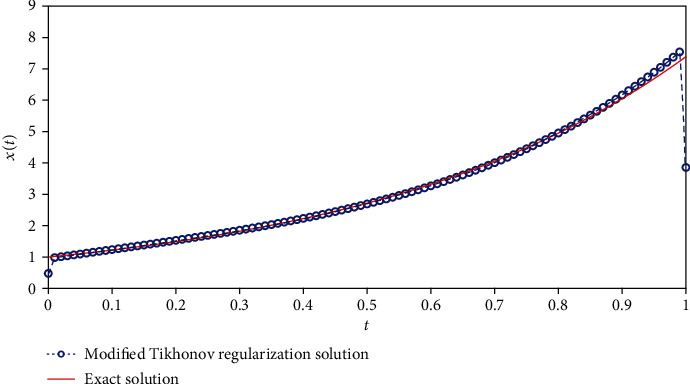
The results of modified regularization method.

The parameters involved in the numerical simulation are the same as the previous example. We also apply the Tikhonov regularization method and our method to solve Equation (34), respectively. The L- curve for Tikhonov regularization method is shown in Figure 6. The corner of L-curve is located at the point for *α* = 4.2365 × 10^−3^.  Figure 7 depicts the comparison between the results obtained by the classical Tikhonov regularization method and the exact solution.  Figure 8 shows the corresponding L-curve for the modified regularization method, and the regularization parameter is 2.7659^∗^*e* − 3.  Figure 9 gives the comparison between the results obtained by the modified regularization method and the exact solution. From Figures 7 and 9, we can conclude that the modified regularization method works better for this problem.

From the previous numerical results, we can conclude that the modified regularization method is effective for the Fredholm integral equation of first kind for the case of input data with noise. Meanwhile, as shown in Section 2, the determination of the initial drug concentration in the optimization problem of drug release based on the multilaminated drug-controlled release devices can be transformed into the solution of the Fredholm integral equation of first kind. So, in the next section, we adopt the proposed method to deal with the optimization problem of drug-controlled release from multilaminated devices.

## 5. Optimization of Drug-Controlled Release from Multilaminated Devices

### 5.1. The Optimization of Initial Drug Concentration

**Figure 10:**
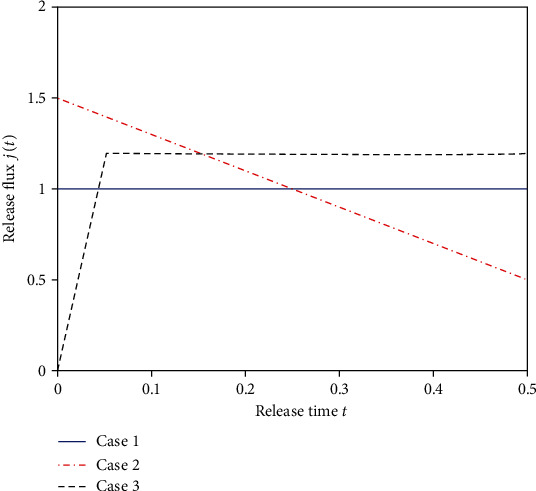
Three different desired flux. Case 1: constant release rate. Case 2: linearly decreasing release rate. Case 3: nonlinear release rate.

The initial drug concentration is an essential parameter in the multilaminated controlled release system, which can affect the drug release greatly. A reasonable initial drug concentration can lead to the drug release with the desired flux. Based on the inverse problem solution scheme, the initial drug concentration can be inverted from the known drug release flux. In the following, we will determine the initial drug concentration for three different cases with the proposed inverse problem solution scheme based on the Tikhonov regularization method (TRM) and the modified regularization method (MRM), respectively. The three different desired release profiles are shown in Figure 10.

#### 5.1.1. Case 1

**Figure 11:**
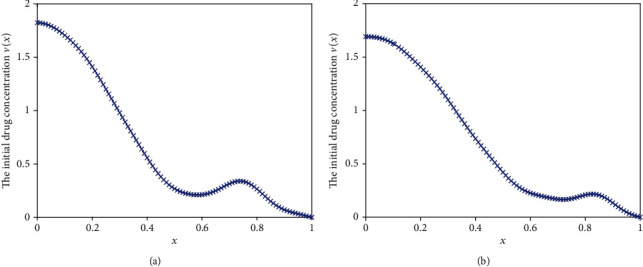
The inverse results. (a) Results obtained by TRM. (b) Results obtained by MRM.

**Figure 12:**
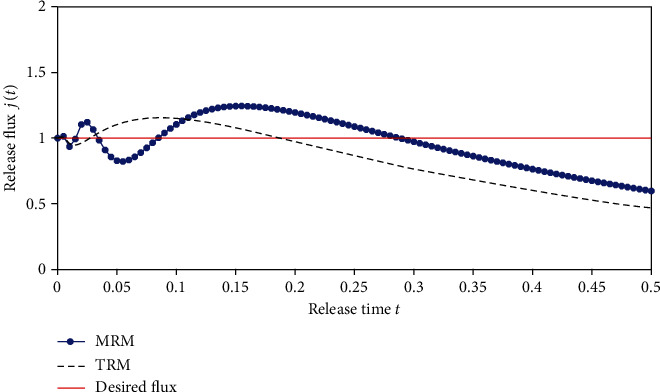
The computational drug release flux based on the inversed initial drug concentration.

We first consider a typical case. In this case, suppose that the desired flux is constant (*j*(*t*) = 1,  0 ≤ *t* ≤ 0.5). For the Fredholm integral equation of the first kind (Equation (12)), set the right-hand side equal to 1, that is, *j*(*t*) = 1. The Tikhonov regularization method and modified regularization method are applied to solve this ill-posed problem. The inverse results are shown in Figure 11, and the computational drug release flux based on the inversed initial drug concentration are depicted in Figure 12. It is seen that from Figure 12, the optimal release profile obtained with TRM remains flatter pattern at initial stage and has a smaller deviation from ideal case. However, bigger error appears as the time increases. The mean square deviation for TRM can reach 0.2174. We can also observe that, for MRM, although the optimized release fluctuates at the initial stage, the deviation from the desired release remains smaller over the entire computation time. Compared with TRM, the mean square deviation for MRM is also relatively less, which is 0.1692.

#### 5.1.2. Case 2

**Figure 13:**
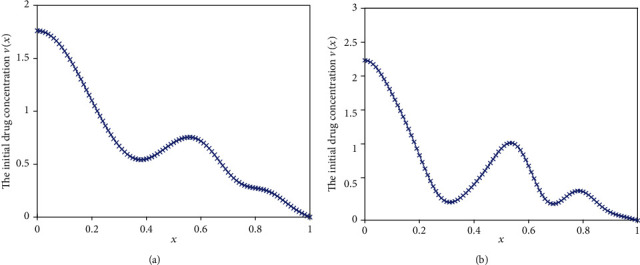
The inverse results. (a) Results obtained by TRM. (b) Results obtained by MRM.

**Figure 14:**
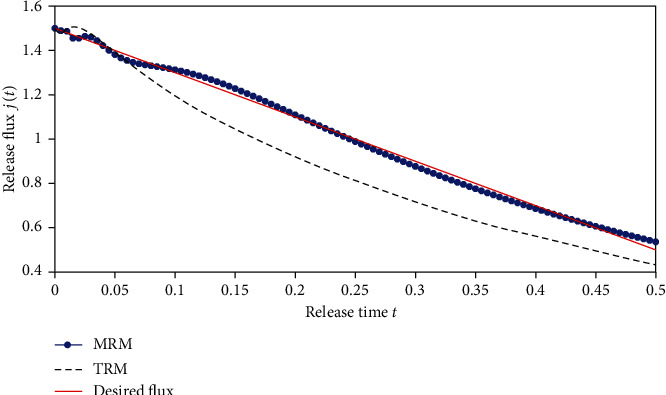
The optimized drug release flux based on the inversed initial drug concentration.

In some cases, we maybe desire to obtain an approximately linearly increasing profile, e.g., to build up a tolerance for the chemical material transmitted. In the following, the ideal release profile is given with the function *j*(*t*) = 1.5 − 2*t*, 0 ≤ *t* ≤ 0.5. The inversed results obtained by using the previous two different regularization methods are shown in Figure 13 and the optimized release profiles are depicted in Figure 14. We can conclude that MRM has obvious advantages over the TRM. In Figure 14, the optimized release profile almost coincides with the desired release flux for MRM, whereas the performance of TRM remains poor. The mean square deviations of two methods are 0.1285 and 0.0164, respectively.

#### 5.1.3. Case 3

Some situations demand a nonlinearly release rate, e.g., linearly increasing follow by a constant release, and without burst. A typical example is the delivery of the some anticancer drug.

For this case, the release rate function is

(35) $$\begin{array}{c} j\left(t\right)=\left\{\begin{array}{cc} 24t & 0\leq t\leq 0.05\\ 1.2 & 0.05<t\leq 0.5 \end{array}\right.. \end{array}$$

**Figure 15:**
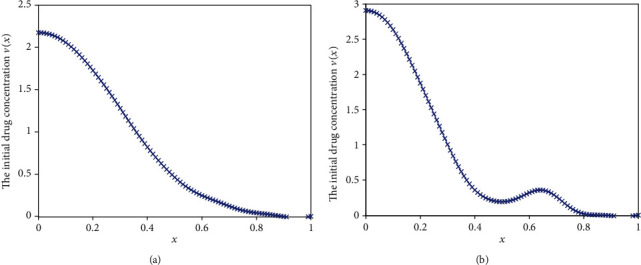
The inverse results. (a) Results obtained by TRM. (b) Results obtained by MRM.

**Figure 16:**
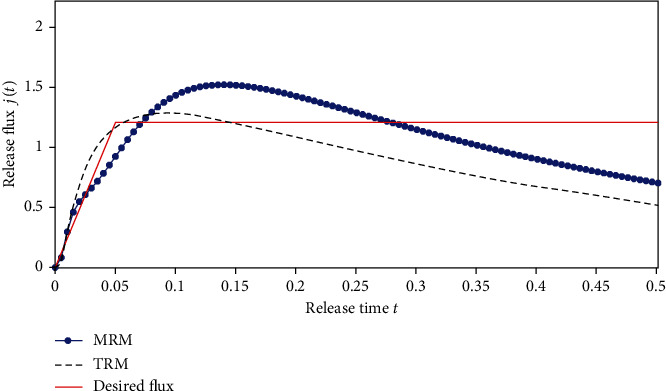
The optimized drug release flux based on the inversed initial drug concentration.

The inversed results obtained by using two different regularization methods are shown in Figure 15 and the optimized release profiles are depicted in Figure 16. From these two figures, we can see that the performance of MRM is better than that of TRM. The mean square deviations of two methods are 0.3019 and 0.2164, respectively.

### 5.2. Antinoise Property Analysis

In practice, we cannot guarantee that the right-hand side of the Fredholm integral equation of the first kind (Equation (12)) is known exactly. Instead, *j*(*t*) usually contains some error, say, *δ* > 0. Therefore, we will consider the initial drug concentration optimization problem for the case of the right-hand side with a perturbed data.

Assume that we know *δ* > 0 and with perturbed data *j*^*δ*^(*t*) satisfying |*j*^*δ*^(*t*) − *j*(*t*)| ≤ *δ*. It is our aim to solve the following perturbed equation.

(36) $$\begin{array}{c} 2\int_{0}^{1}\left[\overset{\infty }{\underset{k=0}{\sum }}{\left(-1\right)}^{k}\left(k+\frac{1}{2}\right)\pi e^{-{\left[\left(k+1/2\right)\pi \right]}^{2}t}\cos\left(k+\frac{1}{2}\right)\pi x\right]v^{\delta }\left(x\right)dx=j^{\delta }\left(t\right). \end{array}$$

**Figure 17:**
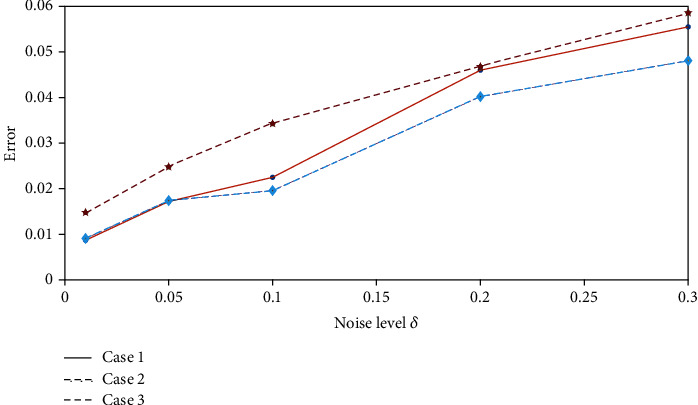
The error against the noise level.

**Figure 18:**
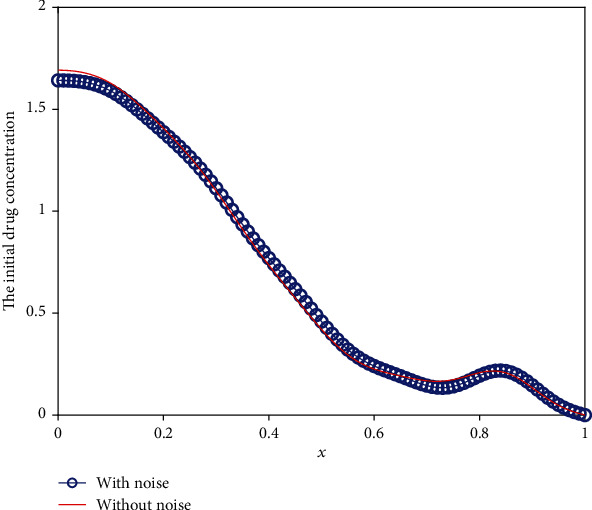
The inverse initial drug concentration for Case 1 with *δ* = 0.1.

**Figure 19:**
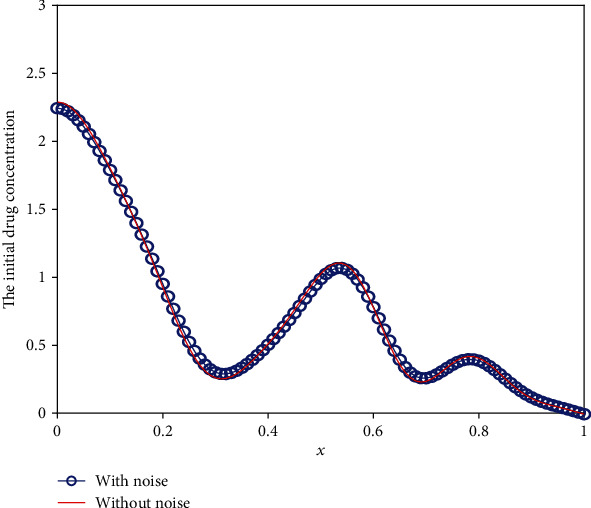
The inverse initial drug concentration for Case 2 with *δ* = 0.1.

**Figure 20:**
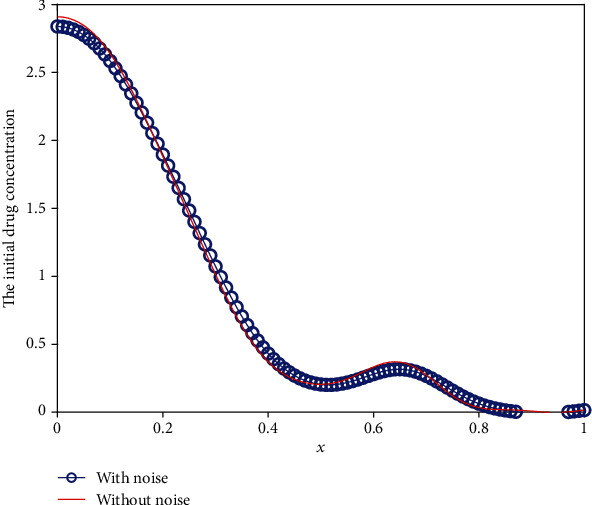
The inverse initial drug concentration for Case 3 with *δ* = 0.1.

We use the modified regularization method to solve the above perturbed equation for three different cases.  Figure 17 shows the error between the inverted results with noise and that without noise against the different noise levels. We can conclude from Figure 17 that the error increases apparently with the increment of noise level. However, even for the noise level of *δ* = 0.3, the results are acceptable. To further show the optimization results intuitively, we list the initial drug concentration optimization results with *δ* = 0.1 for three different cases in Figures 18–20. These three pictures demonstrate that the modified regularization method we proposed can still work well. It means that the MRM has a good antinoise property.

## 6. Conclusion

We have proposed a new viewpoint to solve the optimization problem of drug release based on the multilaminated drug-controlled release device, that is, inverse problem solution scheme. The objective of this paper is to show that the inverse problem solution scheme is effective for the optimization problem of drug release. Based on the inverse problem solution scheme, the optimization problem of drug release can be transformed into the diffusion equation initial value inverse problem and further converted to the Fredholm integral equation of first kind. The solution of Fredholm integral equation of first kind is an ill-posed problem, which have to be solved by suitable regularization method.

To solve this ill-posed problem, we introduce a new regularizing filter function and propose a modified regularization method. The error analysis of the regularized solution obtained by the proposed method is also verified. Furthermore, for three various desired release flux, the modified regularization method is applied to inverse the initial drug concentration. As seen in the examples, the method proposed in this paper has been successful at inverting the initial drug concentration. This demonstrates that the modified regularization approach is well suited to solving this ill-posed problem.

Also shown in this paper is the result that the modified regularization method has a better antinoise property for the initial drug concentration estimation. With 10% noise, the results obtained with the MRM are satisfactory.

In all, the better results obtained in this paper mean that the inverse problem solution scheme exhibits its effectiveness and superiority, for the optimization problem of drug release based on the multilaminated drug-controlled release device, to some extent in both theoretical research and numerical simulation. There is a good potential that the proposed method can be employed to solve more complicated cases, such as multiparameter identification and high-dimensional problem. And this is an important direction for us to face in future.

## Data Availability

The data used to support the findings of this study are available from the corresponding author upon request.
